# DYRK kinases phosphorylate IL-17RA at S801

**DOI:** 10.17161/sjm.v3i3.25722

**Published:** 2026-08-28

**Authors:** Sayed Ala Moududee, Andrew A. Herppich, Mary Kay H. Pflum, Dongxia Ge, Zongbing You

**Affiliations:** 1 Department of Structural & Cellular Biology, Tulane University School of Medicine, New Orleans, LA 70112, USA;; 2 Department of Chemistry, Wayne State University, Detroit, MI 48202, USA;; 3 Department of Orthopaedic Surgery, Tulane University School of Medicine, New Orleans, LA 70112, USA

**Keywords:** IL-17RA, DYRK, in vitro kinase assay, serum starvation, inhibitor

## Abstract

Interleukin-17 (IL-17) receptor A (IL-17RA) promotes inflammatory reactions by activating kinases and transcription regulators. Our previous studies have demonstrated that glycogen synthase kinase 3 (GSK3) phosphorylates IL-17RA at T780. It is not known whether other kinases also phosphorylate IL-17RA. Here, we identified an association between dual-specificity tyrosine-regulated kinases (DYRKs) and IL-17RA. We found that DYRK1B and DYRK4 interacted with and phosphorylated IL-17RA at serine 801 (S801). Overexpression of DYRK1B and DYRK4 enhanced IL-17RA phosphorylation, whereas siRNA-mediated knockdown of DYRK1B or DYRK4 reduced the phosphorylation. Serum starvation of the cultured cells increased expression levels of DYRK1B/4 and IL-17RA phosphorylation, suggesting stress-dependent modulation of receptor modification. Moreover, selective small-molecule inhibitors of DYRKs effectively suppressed IL-17RA phosphorylation at S801. Our findings indicate a link between DYRK family kinases and IL-17RA phosphorylation, providing new potential therapeutic targets in the management of IL-17-mediated diseases.

## Introduction

Interleukin-17 (IL-17 or IL-17A) represents a family of pro-inflammatory cytokines constituting a key mechanism in host defense and pathogenesis of chronic inflammatory disorders. Several studies have characterized the transcriptional, translational, and regulatory mechanisms of IL-17 to modulate its expression or inhibit its signaling pathways to develop therapeutic strategies for immunological disorders and IL-17–associated cancers [1–3]. IL-17A is the first member of the IL-17 family to be identified and remains the most extensively studied and biologically significant cytokine within this group, which consists of six structurally related members: IL-17A, IL-17B, IL-17C, IL-17D, IL-17E (also known as IL-25), and IL-17F [3]. It is also termed cytotoxic T-lymphocyte-associated antigen 8 (CTLA-8) [4]. IL-17A contributes to host defense against fungal and extra-cellular bacterial pathogens, while its dysregulated activity has been linked to immune-mediated conditions such as rheumatoid arthritis, psoriasis, multiple sclerosis, inflammatory bowel diseases [5–7], and anxiety-related psychiatric conditions [8–13]. Beyond its critical role in host defense and autoimmune disorders, IL-17 has emerged as a crucial mediator in tumor biology. Elevated IL-17 levels within the tumor microenvironment enhance chronic inflammation, angiogenesis, and recruitment of immunosuppressive cells, thereby facilitating tumor growth and metastasis [14, 15]. A recent study showed that IL-17A levels were increased in 59 diseases involving cardiovascular, metabolic, cancer, psychiatric, autoimmune, infection, and pediatric categories [16]. On the other hand, in certain contexts, IL-17 promotes anti-tumor immunity by stimulating cytotoxic T-cell responses and promoting tumor cell clearance [17, 18]. The dual, context-dependent roles of IL-17 make it a double-edged sword in cancer. Accordingly, therapeutic strategies targeting IL-17 or its signaling pathways are being elucidated in cancer immunotherapy, both as standalone interventions and in combination with immune checkpoint inhibitors [19, 20] to modulate the tumor microenvironment and facilitate anti-tumor immunity while limiting tumor-promoting inflammation [21].

Recent evidence indicates that IL-17RA-mediated signaling intersects with intracellular kinases that regulate cell proliferation, survival, and transcriptional programs [22]. We have previously identified eight amino acid residues of IL-17RA that are potentially phosphorylated, and glycogen synthase kinase 3 (GSK3) constitutively binds to and phosphorylates IL-17RA at T780 [23]. IL-17RA phosphorylation at T780 leads to ubiquitination mediated by F-box and WD repeat domain-containing 11 (FBXW11) E3 ligase, resulting in proteasome-mediated degradation of IL-17RA [24]. Recently, a kinome-wide study [25] developed an algorithm and predicted the eight IL-17RA phosphorylation residues that we previously identified [23]. Among the potential kinases, the dual-specificity tyrosine-phosphorylation-regulated kinase (DYRK) family is predicted to phosphorylate IL-17RA at serine 801 (S801). The term “dual specificity” refers to the ability of DYRK kinases to phosphorylate tyrosine and serine/threonine residues. Autophosphorylation of a tyrosine residue within their activation loop is required for full activation, which is crucial for enabling their serine/threonine kinase activity. Once activated, the enzymes lose their tyrosine specificity, and their enzymatic activity is confined to serine and threonine residues [26]. DYRK family has five members, including DYRK1A, DYRK1B, DYRK2, DYRK3, and DYRK4 [27]. The roles of DYRK kinases in IL-17RA phosphorylation remain unexplored. In the present study, we investigated the interactions between all members of the DYRK family and IL-17RA and their effects on IL-17RA kinase activity. Our results revealed that DYRK1B and DYRK4 bound to and phosphorylated IL-17RA at S801. Overexpression of the two kinases increased the phosphorylation, whereas knockdown of either kinase reduced the phosphorylation. Furthermore, serum deprivation of the cultured cells induced expression of DYRK1B and DYRK4 and simultaneously increased phosphorylation of IL-17RA at S801. In addition, selective inhibitors of DYRKs reduced IL-17RA phosphorylation. These findings suggest that DYRK1B and DYRK4 are kinases that phosphorylate IL-17RA at S801. DYRK1B and DYRK4 may play a role in regulating IL-17 signaling, which can be targeted to develop new avenues for therapeutic intervention in diseases associated with dysregulated IL-17 activities.

## Materials and Methods

### Mammalian cell culture and plasmids

Human embryonic kidney cell line (HEK293T) was purchased from the American Type Culture Collection (ATCC, Manassas, VA, USA). A stable cell line (293-IL-17RA) overexpressing exogenous FLAG-IL-17RA was established in our lab [23]. HEK293T and 293-IL-17RA cell lines were maintained in high-glucose Dulbecco’s Modified Eagle Medium (DMEM) (Genesee Scientific, San Diego, CA, USA, cat# 25-500) supplied with 10% fetal bovine serum (FBS, ATCC, Manassas, VA, USA, cat# 30-2022). 293-IL-17RA cells were treated with 500 μg/ml neomycin sulfate (Millipore Sigma, Cat# 1405-10-3) to select the cells stably expressing the neomycin-resistant gene and FLAG-tagged human full-length IL-17RA. MYC-HIS-IL-17RA plasmid was a gift from Dr. Reen Wu (University of California-Davis) [28]. Human full-length FLAG-IL-17RA plasmid was acquired from Dr. Xiaoxia Li’s lab [29]. FLAG-DYRK1A, FLAG-DYRK1B, FLAG-DYRK2, FLAG-DYRK3, and FLAG-DYRK4 full-length plasmids were obtained from Dr. Yoshihiko Miyata, Kyoto University [27].

### Transfection of small interfering RNA (siRNA)

Human DYRK1B siRNA (cat# sc-77210), DYRK3 siRNA (cat# sc-39010), DYRK4 siRNA (cat# sc-77231), and nontargeting control siRNA (cat# sc-37007) were purchased from Santa Cruz Biotechnology (Dallas, TX, USA). Cells were transfected with siRNAs when their confluency reached around 70–80%. Transfection was performed using jetPRIME transfection reagent (Polyplus, New York, NY, USA, cat# 114-15). Briefly, 40 nanomoles of control siRNA or gene-specific siRNA were diluted in 500 μL jetPRIME buffer, vortexed for 10 seconds, and spun down transiently. Six μl of jetPRIME reagent was added, vortexed for 1 second, spun down briefly, and incubated at room temperature for 10 minutes to allow complex formation. The mixture was added dropwise onto the cells evenly in serum-containing medium. After transfection, the cells were incubated for 24 hours, then the culture medium was replaced with fresh medium, and subsequent treatments were conducted accordingly.

### Co-immunoprecipitation (co-IP)

HEK293T cells were seeded at a density of 4.5 × 10^6^ cells per 10-cm dish approximately 18–20 h before co-transfection with MYC-HIS-IL-17RA and FLAG-DYRK1A, FLAG-DYRK1B, FLAG-DYRK2, FLAG-DYRK3, or FLAG-DYRK4 plasmids. 7 mL of DMEM (with 10% FBS) was added to 10-cm dishes. Transient transfection of the plasmids was conducted using jetPRIME. Briefly, 3 μg of MYC-HIS-IL-17RA and 3 μg of one of the DYRK plasmids were diluted in 500 μL jetPRIME buffer, vortexed for 10 seconds, and spun down transiently. 12 μl of jetPRIME reagent was added, vortexed for 1 second, spun down briefly, and incubated at room temperature for 10 minutes to allow complex formation. The mixture was added dropwise onto the cells evenly in serum-containing medium. After transfection, the cells were incubated for 24 hours, and then the culture medium was replaced with fresh medium. Forty-eight hours post-transfection, protein was extracted with an IP lysis buffer (50 mM Tris-HCl (pH 7.5), 0.5% Nonidet P-40, 1 mM EDTA, and 150 mM NaCl). IP lysis buffer was freshly supplemented with 1× protease inhibitor cocktail (PIC, Millipore Sigma, Saint Louis, MO, USA, cat# P8849) and 1 mM 1,4-dithiothreitol (DTT, Thermo Fisher, Lenexa, KS, USA, cat# R0861). An appropriate amount of lysis buffer was added and vortexed vigorously for 5 s, followed by incubation on ice for 25 min with vortexing every 5 min. Cell debris was removed by centrifugation at 13,000× *g* at 4 °C for 20 min. The supernatant was transferred into a new sterile centrifuge tube, and the protein concentration was quantified with the Bradford assay[30]. Immunoprecipitation was carried out by mixing 600–800 μg of whole-cell lysate and 1–2 μg of anti-FLAG M2 antibody (Sigma Millipore, St. Louis, MO, USA, cat# F3165) or 1–2 μg of anti-c-MYC antibody (Santa Cruz Biotechnology, Dallas, TX, USA, cat# sc-40). One to two hours later, 18–20 μL of 75% (*v*/*v*) protein A agarose resin beads (Genesee Scientific, San Diego, CA, USA, cat# 20-525) were added to each sample, and incubation was maintained at 4 °C overnight with rotation. The beads were washed 3 times with 1 mL of pre-chilled IP lysis buffer (freshly supplemented with 1 mM DTT and 1X PIC). The precipitated proteins were eluted in 20 μL of 3X sample loading buffer with boiling at 100 °C for 5 min. The samples were subjected to Western blot analysis.

### In vitro kinase assay

293-IL-17RA cells were seeded at a density of 4.5 × 10^6^ cells per 10-cm dish, containing 7 mL of DMEM supplemented with 10% FBS and cultured for 24 hours. After 24 hours, the medium was replaced with fresh complete medium, and cells were harvested 48 h post-transfection. For cell lysis under nondenaturing conditions, cells were washed once with ice-cold phosphate-buffered saline (PBS), collected into new sterile microcentrifuge tubes, and kept on ice. After centrifugation at 10,000 rpm for 1 min, the supernatant was discarded, and the pellets were lysed in buffer containing 50 mM Tris-HCl (pH 7.5), 0.5% NP-40, 150 mM NaCl, 1 mM EDTA, 1 mM DTT, and 1x PIC. Lysates were vortexed intermittently on ice and clarified by centrifugation at 10,000 rpm for 20 min, after which the supernatant was collected as the cell lysate. Protein concentration was quantified, and approximately 4 mg of total protein was incubated with 1.5 μg of anti-FLAG antibody for 1 h at 4°C with rotation. After 1 h of incubation, protein A agarose beads (20 μl of 50% bead slurry) were added and incubated with gentle rocking overnight at 4°C. After incubation, the mixture was centrifuged for 1 minute at 4°C. The supernatant was discarded, and the bead pellet was washed twice with 1 mL of the cell lysis buffer, keeping it on ice during the washes. Then, the pellet was washed twice with 500 μl 1X kinase buffer and subjected to an *in vitro* kinase assay in kinase buffer (25 mM Tris (pH 7.5), 5 mM β-glycerophosphate, 2 mM DTT, 0.1 mM Na_3_VO_4_, 10 mM MgCl_2_) supplemented with 2 mM ATP-polyamine-biotin (MedChemExpress, USA, cat# HY-D0183) or ATP-biotin synthesized in Dr. Mary Kay Pflum’s laboratory [31] (of note, the two ATP analogs yielded similar results) and 2 μl of recombinant DYRK1B (cat# D09-10BG-05, SignalChem/Sino Biological Inc., Richmond, BC Canada V6V 2J2) or 2 μl of recombinant DYRK4 (cat# D12-10G-05, SignalChem/Sino Biological Inc., Richmond, BC Canada V6V 2J2)) at 31°C for 2 hours. Reactions were terminated with 4x sample loading buffer, heated at 95–100°C, resolved by sodium dodecyl sulfate-polyacrylamide gel electrophoresis (SDS-PAGE), and transferred to polyvinylidene difluoride (PVDF) membranes. Membranes were blocked with bovine serum albumin-based blocking buffers, incubated with Avidin-Fluor 680 (Tymora Analytical Operations, West Lafayette, IN, 1:1000 dilution), washed, and scanned to detect kinase-catalyzed biotinylation.

### Cell treatment

293-IL-17RA cells were treated with small molecule inhibitors HTH-01-091 (MCE cat# HY-122665, inhibiting maternal embryonic leucine zipper kinase at IC_50_ of 10.5 nM and inhibiting DYRK4 at IC_50_ of 41.8 nM) and DYRK1A-IN-4 (MCE cat# HY-147066, inhibiting DYRK1A at IC_50_ of 2 nM and DYRK2 at IC_50_ of 6 nM) at final concentrations of 0.1 μM, 0.5 μM, and 1 μM, containing medium with 10% or 0.1% FBS for the indicated time periods. Compounds were prepared as dimethyl sulfoxide (DMSO) stocks, and control cells received an equivalent volume of DMSO.

### Western blot analysis

**Figure 1:**
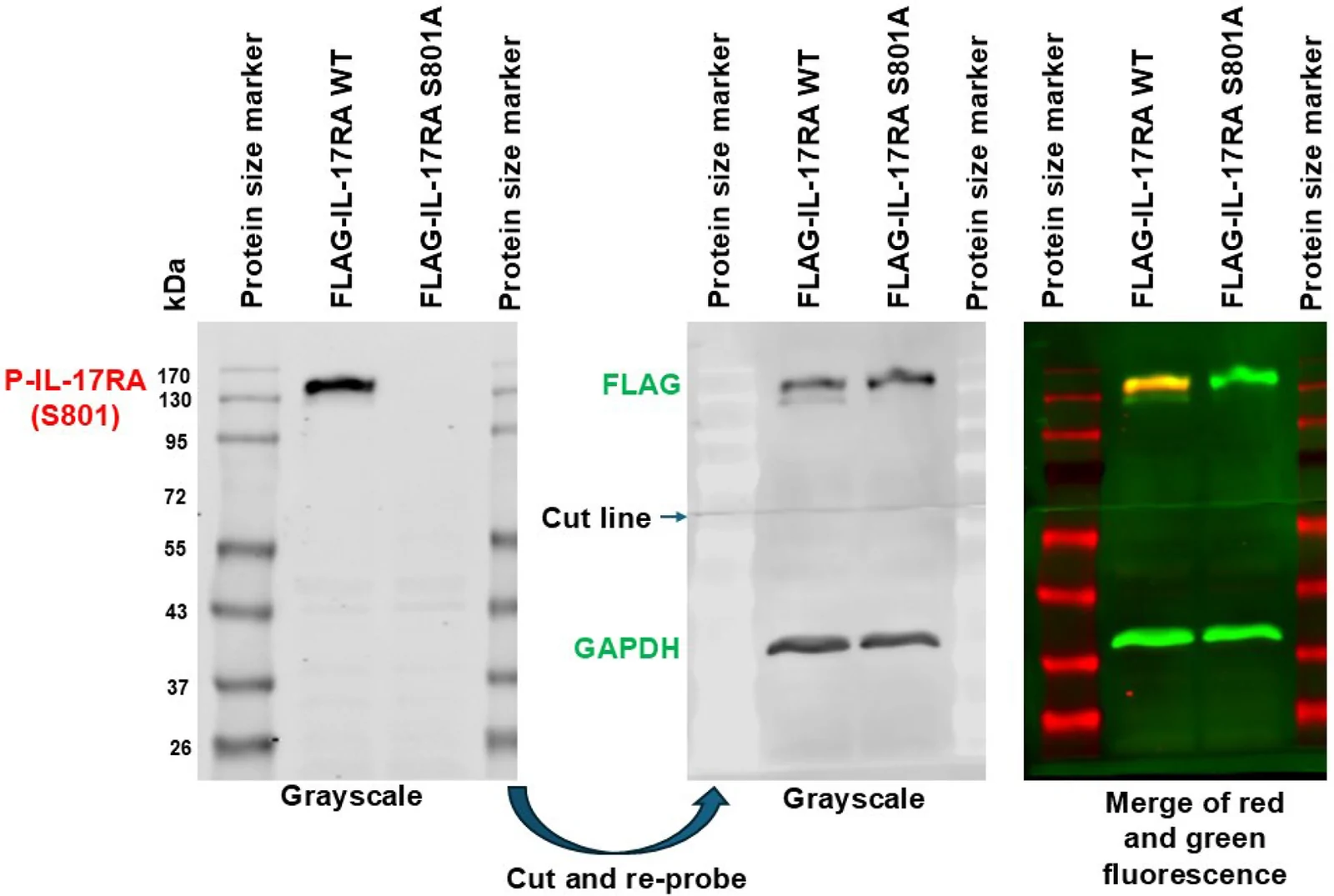
Characterization of rabbit anti-P-IL-17RA (S801) polyclonal antibody. Wild-type (WT) FLAG-IL-17RA (FLAG-IL-17RA WT) and FLAG-IL-17RA S801A site-mutant proteins were expressed in 293 cells. Western blot analysis was first performed to detect P-IL-17RA (S801) using rabbit anti-P-IL-17RA (S801) antibody and an anti-rabbit secondary antibody conjugated to red fluorescence, which showed a clear band in the FLAG-IL-17RA WT but not the FLAG-IL-17RA S801 mutant; then, the same membrane was cut into upper and lower portions to detect FLAG tag and GAPDH using mouse anti-FLAG or mouse anti-GAPDH antibodies and an anti-mouse secondary antibody conjugated to green fluorescence, respectively. When the red and green fluorescence were merged, the overlap occurred in the upper band of the two green bands of FLAG-IL-17RA WT, indicating a slightly higher molecular weight of the P-IL-17RA than the non-phosphorylated IL-17RA.

Cells were washed with PBS and lysed with radioimmunoprecipitation assay (RIPA) buffer (50 mM sodium fluoride, 0.5% NP-40, 10 mM sodium phosphate monobasic, 150 mM sodium chloride, 25 mM Tris (pH 8.0), 2 mM ethylenediaminetetraacetic (EDTA), and 0.2 mM sodium vanadate) with a freshly supplemented 1X PIC. After incubation on ice for 20 min, the cell lysate was centrifuged at 13,000× *g* for 20 min at 4 °C. The supernatant of the whole-cell lysates was transferred into a new sterile microcentrifuge tube and boiled with 3x sample loading buffer (208.1 mM sodium dodecyl sulfate, 30% glycerol, 187.5 mM Tris (pH 6.8), 15% β-mercapto-ethanol, and 14.9 mM bromophenol blue) at 100 °C for 5–10 min. Approximately 60–120 μg of total protein was subjected to SDS-PAGE and transblotted to PVDF membranes. The blots were blocked with 2.5% bovine serum albumin (Millipore Sigma, cat# A3294) solution containing 0.02% sodium azide (Millipore Sigma, cat# S2002) and probed with the following primary antibodies at room temperature for 1 h or at 4 °C overnight: rabbit anti-phospho-IL-17RA (S801) polyclonal antibodies (1:200 dilution) developed in our lab using a phosphorylated antigen peptide (C-YISRS(pS)PQPPEG) through a contract with Abmart Inc (Shanghai, China), and the specificity of the antibody was confirmed using FLAG-IL-17RA S801A site-mutant protein (Figure 1); anti-GAPDH (Millipore Sigma, cat# MAB374, 1:5000 dilution), anti-FLAG M2 (Millipore Sigma, cat# F3165, 1:20,000 dilution), anti-IL-17RA (Santa Cruz Biotechnology, cat# sc-376374, 1:500 dilution), antic-MYC (Santa Cruz Biotechnology, cat# sc-40, 1:1000 dilution), anti-DYRK1A (ABclonal, cat#A0595, 1:500 dilution), anti-DYRK1B (ThermoFisher Scientific, cat#PA5-85318, 1:500 dilution), anti-DYRK2 (ABclonal, cat#A7012, 1:500 dilution), anti-DYRK3 (Santa Cruz Biotechnology, cat#sc-390732, 1:500 dilution), and anti-DYRK4 (ThermoFisher Scientific, cat#PA5-55758, 1:500 dilution). Li-Cor IRDye680- and IRDye800-conjugated secondary antibodies (Lincoln, NE, USA) were incubated at room temperature for 60 min at dilutions of 1:10,000 and 1:5,000, respectively. The blots were scanned with a Li-COR Odyssey 9120 Digital Imaging system (Lincoln, NE, USA). When necessary, the blots were stripped with a stripping buffer (62 mM Tris-HCl (pH 6.7), 2% SDS, and 100 mM β-mercapto- ethanol) and re-scanned to confirm stripping efficiency before probing for another antigen of interest. Adobe Illustrator 2025 (San Jose, CA, USA) was utilized to organize the images.

## Results

### 1. DYRKs interact and phosphorylate IL-17RA

**Figure 2:**
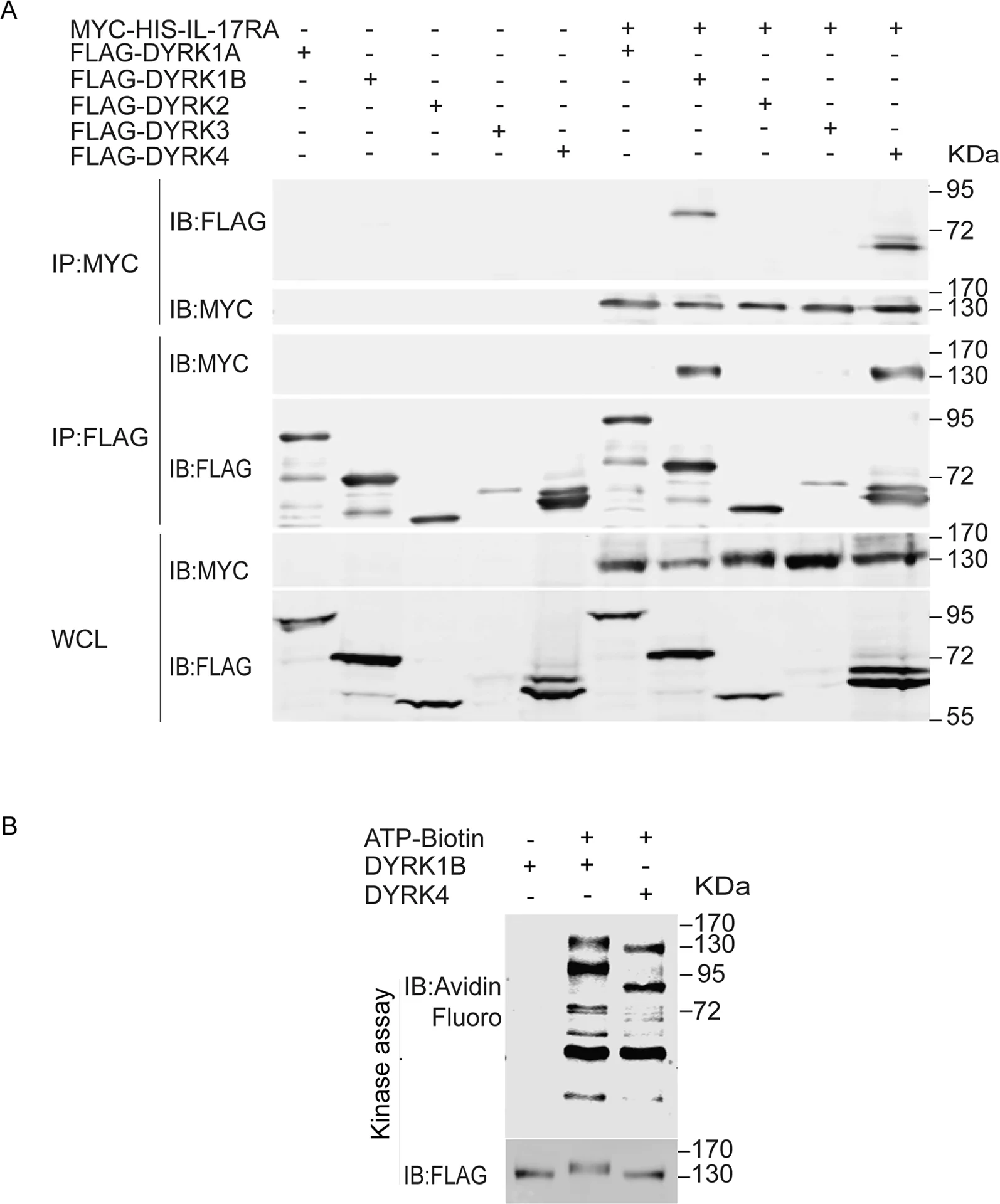
DYRKs bind to and phosphorylate IL-17RA. **A.** HEK293T cells were co-transfected with MYC-HIS-IL-17RA, FLAG-DYRK1A, FLAG-DYRK1B, FLAG-DYRK2, FLAG-DYRK3, and FLAG-DYRK4. WCL, whole-cell lysate. **B.** 293-IL-17RA cells were cultured and lysed, and FLAG-IL-17RA was immunoprecipitated using 1.5 μg of anti-FLAG antibodies and used as the substrate for DYRK1B or DYRK4 in *in vitro* kinase assays. Biotinylated proteins were detected using Avidin-Fluor 680. Of note, the protein bands smaller than 130 kDa might be degraded FLAG-IL-17RA fragments or other proteins that were co-immunoprecipitated with Flag-IL-17RA and biotinylated by the kinases. The experiments were repeated three times independently.

To investigate whether DYRKs could phosphorylate IL-17RA or not, we performed co-IP assays to screen all members of the DYRK family that might bind to IL-17RA. Myc-His tagged IL-17RA was co-transfected with FLAG-tagged DYRK1A, DYRK1B, DYRK2, DYRK3, or DYRK4 into HEK293T cells. Our reciprocal co-IP results showed that IL-17RA interacted with DYRK1B and DYRK4, but not with DYRK1A, DYRK2, or DYRK3, as shown by the fact that Myc-His-tagged IL-17RA was able to pull down FLAG-tagged DYRK1B and DYRK4, and vice versa, but not DYRK1A, DYRK2, or DYRK3 (Figure 2A). To check if DYRK1B and DYRK4 could phosphorylate IL-17RA, we performed *in vitro* kinase assays in the presence of either ATP-polyamine-biotin or ATP-Biotin as a phosphate donor in 293-IL-17RA cells. We immunoprecipitated FLAG-IL-17RA from 293-IL-17RA cells and used it as the substrate. Both recombinant DYRK1B and DYRK4 efficiently phosphorylated IL-17RA in the presence of ATP-polyamine-biotin or ATP-Biotin, which was detected by Avidin–Fluor 680 fluorescence. In contrast, no fluorescence signal was detected in the absence of ATP-polyamine-biotin or ATP–Biotin. Subsequently, we probed the same membrane with anti-FLAG antibody to show equal presence of FLAG-IL-17RA (Figure 2B).

### 2. DYRK1B and DYRK4 expression controls phosphorylation of IL-17RA

**Figure 3:**
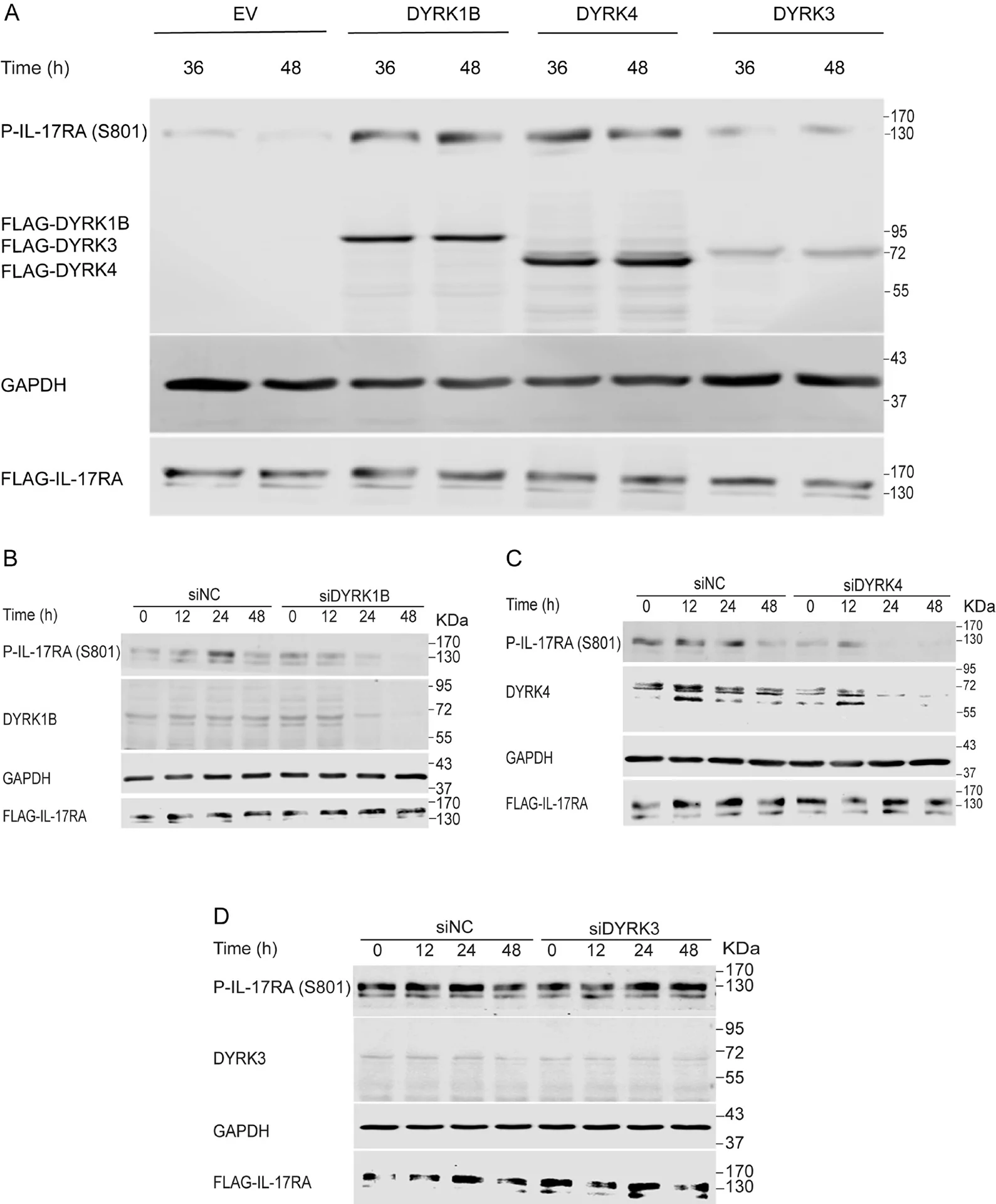
DYRK1B and DYRK4 expression controls phosphorylation of IL-17RA. **A.** Empty vector (EV), FLAG-DYRK1B, FLAG-DYRK3, or FLAG-DYRK4 were transfected into the 293-IL-17RA cell line. **(B, C, D).** 293-IL-17RA cells were transfected with control siRNA and siRNAs targeting DYRK1B, DYRK3, or DYRK4. Endogenous DYRK1B, DYRK3, and DYRK4 levels were detected with the indicated antibodies. The experiments were independently repeated three times.

To further investigate the roles of DYRK1B and DYRK4 in IL-17RA phosphorylation, we transfected 293-IL-17RA cells with FLAG-DYRK1B, DYRK3, or DYRK4. We found that overexpression of DYRK1B and DYRK4 led to a pronounced increase in phosphorylation of IL-17RA at S801, while minimal P-IL-17RA levels were observed in the cells transfected with DYRK3 or empty vector (EV) (Figure 3A). Phosphorylation of S801 was chosen based on a software prediction of the phosphorylation site. Conversely, siRNA-mediated knockdown of DYRK1B or DYRK4 remarkably reduced IL-17RA phosphorylation at S801 (Figure 3B and 3C), whereas siRNA-mediated knockdown of DYRK3 did not show any effects (Figure 3D).

### 3. Serum starvation increases IL-17RA phosphorylation at S801

**Figure 4:**
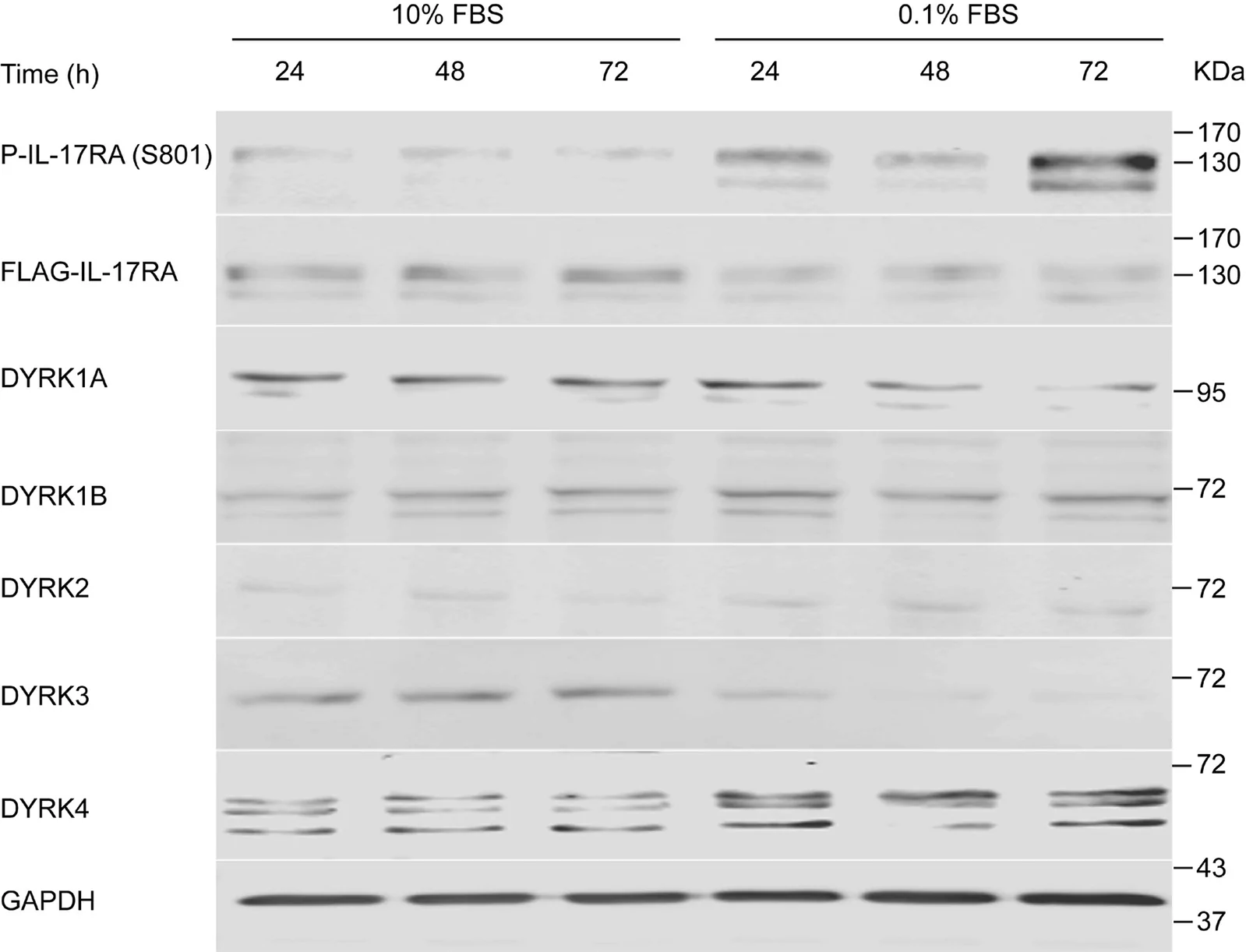
Serum starvation induces DYRK1B and DYRK4 expression and increases phosphorylation of IL-17RA at S801 in 293-IL-17RA cells. Cells were cultured in 10% FBS and 0.1% FBS-containing medium, and cell lysates were harvested after 24, 48, and 72 h, respectively. The experiments were repeated three times.

Previous studies have shown that serum starvation upregulates DYRK1B expression in cancer cells, which induces cell-cycle arrest as a stress-adaptive survival mechanism [32, 33]. To check whether expression of DYRK1B and other DYRKs might be affected by serum starvation and whether IL-17RA phosphorylation might be affected subsequently, 293-IL-17RA cells were subjected to a culture medium with10% FBS (a routine complete culture medium) or serum starvation (0.1% FBS-containing medium) for 24, 48, and 72 hours, mimicking deprivation of growth factors and nutrients. Under serum starvation conditions, we observed a substantial increase in expression levels of DYRK1B and DYRK4 but not DYRK1A, DYRK2, or DYRK3, compared to the cells maintained in the complete culture medium. Simultaneously, phosphorylation of IL-17RA at S801 was dramatically elevated (Figure 4).

### 4. DYRK inhibition suppresses IL-17RA phosphorylation at S801

**Figure 5:**
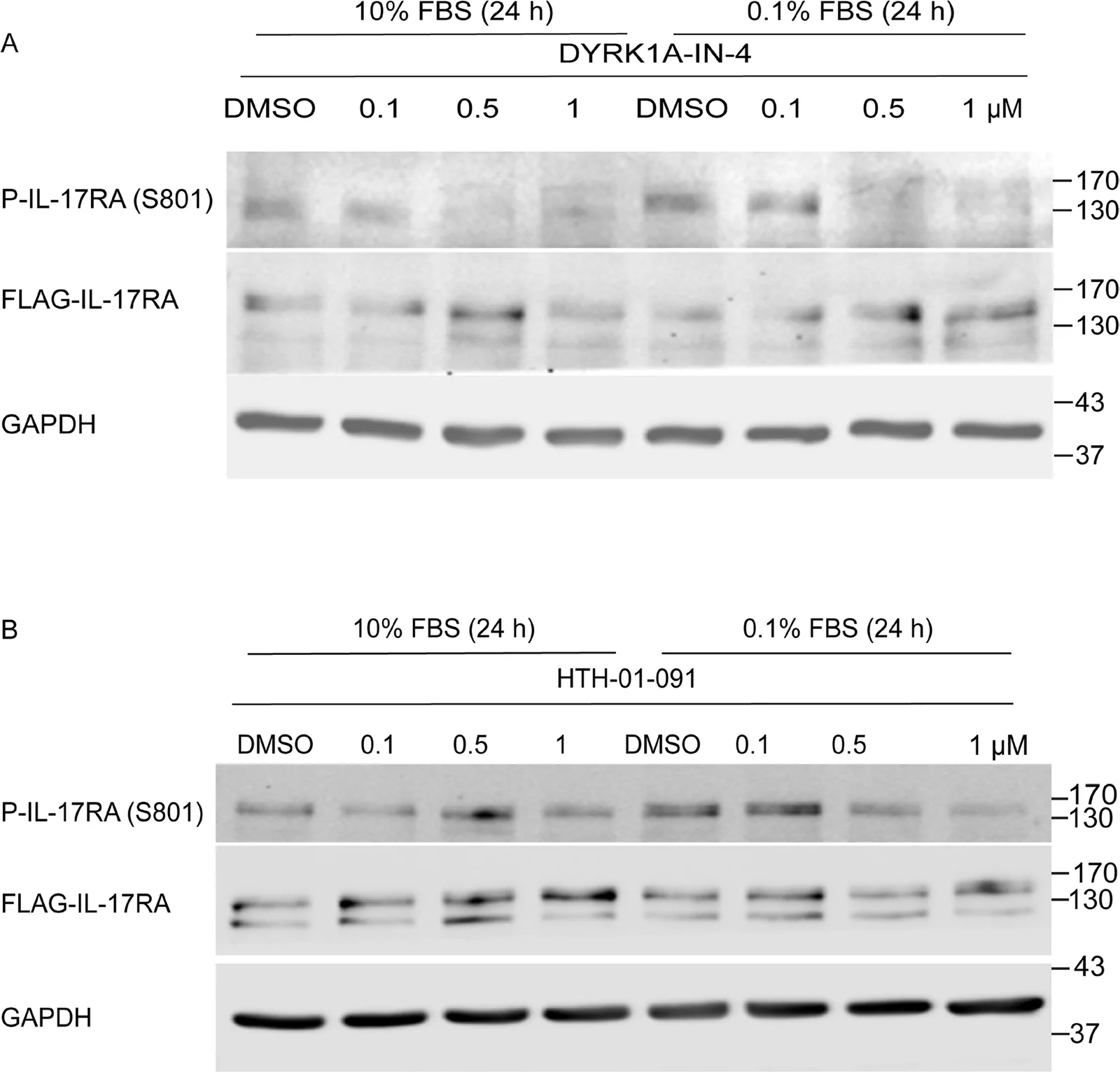
DYRK inhibition suppresses phosphorylation of IL-17RA at S801. **(A, B).** 293-IL-17RA cells were cultured in media containing 10% FBS and 0.1% FBS and treated with DYRK1A-IN-4 and HTH-01-091 at different doses for 24 h.

Specific inhibitors against DYRKs are not available. Several small-molecule kinase inhibitors may selectively inhibit DYRKs, such as DYRK1A-IN-4 (inhibiting DYRK1A at an IC_50_ of 2 nM and DYRK2 at an IC_50_ of 6 nM) and HTH-01-091 (inhibiting DYRK4 at an IC_50_ of 41.8 nM, which is a selective inhibitor). 293-IL-17RA cells were cultured in media containing 10% or 0.1% FBS and treated with DYRK1A-IN-4 and HTH-01-091 at three doses for 24 h. We found that both inhibitors decreased IL-17RA phosphorylation at S801 in the cells cultured with 10% FBS and 0.1% FBS (Figure 5A and 5B).

## Discussion

The diverse interactions between IL-17 cytokines and their receptors have prompted extensive research to demonstrate the expression profiles, cellular localization, and context-dependent functions of individual IL-17 family members and their cognate receptors [34–36]. Among these, IL-17RA plays a key role in mediating downstream signaling cascades that drive the expression of pro-inflammatory cytokines and chemokines [37]. In addition, most research to date has focused on dissecting the regulation of downstream IL-17 receptor signaling and its impact on physiology and disease [38–41]. Indeed, proteomics studies have revealed that kinases such as TANK binding kinase 1 (TBK1) and IκB kinase epsilon (IKKε) are key elements in IL-17RA signaling complexes. These kinases are significantly activated when IL-17 binds to its receptors and play a crucial role in the regulation of downstream pathways [42–44]. While previous studies have identified kinases such as TBK1 and IKKε as integral components of the IL-17RA signaling complex, suggesting that kinase recruitment modulates IL-17–driven responses, to the best of our knowledge, the potential link between DYRK family kinases and IL-17RA has not been reported previously. In this study, we identified DYRK1B and DYRK4 as kinases that phosphorylate IL-17RA at S801, revealing a previously unrecognized connection between DYRK family kinases and IL-17 receptor signaling. Our reciprocal co-immunoprecipitation experiments demonstrate that DYRK1B and DYRK4, but not other DYRK family members, interact with IL-17RA, suggesting a potential regulatory mechanism whereby these kinases might directly modulate receptor functions.

DYRK family of serine/threonine protein kinases regulates cellular signaling through protein phosphorylation [45]. Recent evidence has shown that DYRK1B directly phosphorylates specific protein substrates, including transcription factors, thereby modulating downstream signaling pathways involved in metabolic regulation, cell differentiation, and stress responses [46, 47]. Notably, DYRK1B-mediated phosphorylation of the transcription factor Forkhead Box O1 (FOXO1) induces its nuclear localization and transcriptional activity, thus linking the kinase activity of DYRK1B to functional metabolic outcomes [46]. On the other hand, DYRK4 is still poorly characterized; biochemical and cellular studies reveal that DYRK4 exhibits intrinsic kinase activity and promotes the regulation of phosphorylation-dependent signaling pathways linked with innate immune responses and autophagy [47, 48]. In the current study, our observation of DYRK1B and DYRK4 kinases as binding partners of IL-17RA suggests an intriguing possibility that IL-17RA may serve as a bona fide DYRK substrate. To test this hypothesis, we performed an *in vitro* kinase assay to determine whether DYRK1B and DYRK4 directly phosphorylate IL-17RA, using Flag-IL-17RA substrate that was immunoprecipitated from the protein lysate of the 293-IL-17RA cell line. Both DYRK1B and DYRK4 efficiently phosphorylated IL-17RA, as indicated by detection of specific Avidin–Fluor 680 signals. Our Co-IP and *in vitro* kinase assays demonstrate that DYRK1B and DYRK4 are not only associated with but also directly phosphorylate IL-17RA.

Our lab has previously identified multiple phosphorylation sites of IL-17RA and confirmed IL-17RA phosphorylation at T780 by GSK3, thereby promoting FBXW11-mediated ubiquitination and proteasome-mediated degradation of IL-17RA [23, 24]. In the present study, we identified that DYRK1B and DYRK4 phosphorylated IL-17RA at S801 using a site-specific anti-phospho-IL-17RA antibody. S801 is a site located at the C-terminal cytoplasmic tail of IL-17RA that plays a key role in downstream signal integration [3, 29, 49]. Overexpression of DYRK1B or DYRK4 enhanced phosphorylation of IL-17RA at S801. In contrast, siRNA-mediated knockdown of DYRK1B or DYRK4 decreased the phosphorylation, supporting the conclusion that DYRK1B and DYRK4 function as kinases to phosphorylate IL-17RA. However, the biological consequences of S801 phosphorylation are not clear, which shall be investigated in future studies.

Our serum starvation experiments revealed that cellular stress increased IL-17RA phosphorylation. Previous reports showed that serum deprivation upregulated DYRK1B expression as an adaptive response to metabolic and mitogenic stress [50, 51]. Here, we observed upregulation of both DYRK1B and DYRK4 under low-serum conditions, accompanied by enhanced phosphorylation of IL-17RA at S801. Selective DYRK inhibitors constitute powerful pharmacological probes for evaluating the functional consequences of DYRK-dependent signaling and are being actively pursued as modulators of kinase-driven pathologies, including immune-mediated inflammatory disorders [52]. In this study, we found that DYRK1B and DYRK4 phosphorylated IL-17RA at S801, a modification that was suppressed by selective DYRK inhibitors such as DYRK1A-IN-4 and HTH-01-091, indicating that IL-17RA phosphorylation at S801 may be blocked with DYRK inhibitors. Whether this pharmacological intervention has any therapeutic potential requires further studies, which are not clear currently.

## Conclusion

In summary, our present study revealed that, among the five DYRK family members, only DYRK1B and DYRK4 interact with IL-17RA and can directly phosphorylate this receptor at S801. Serum starvation can increase DYRK1B and DYRK4 expression and simultaneously increase IL-17RA phosphorylation. Selective DYRK inhibitors can reduce IL-17RA phosphorylation, suggesting potential pharmacological intervention of IL-17RA phosphorylation. However, further studies are needed to understand the biological consequences of IL-17RA phosphorylation by DYRK1B and DYRK4 and to explore its relevance in the pathogenesis of IL-17-associated diseases.

## Data Availability

The data that support the findings of this study are present in the manuscript, and further information is available from the corresponding author upon request.

## References

[1] Beringer A, Noack M, Miossec P: IL-17 in Chronic Inflammation: From Discovery to Targeting. Trends Mol Med 2016, 22(3):230–241. doi:10.1016/j.molmed.2016.01.001:26837266

[2] Maione F: Commentary: IL-17 in Chronic Inflammation: From Discovery to Targeting. Front Pharmacol 2016, 7:250. doi:10.3389/fphar.2016.00250. 27561214 PMC4980561

[3] Gaffen SL: Structure and signalling in the IL-17 receptor family. Nat Rev Immunol 2009, 9(8):556–567. doi:10.1038/nri2586. 19575028 PMC2821718

[4] Rouvier E, Luciani MF, Mattei MG, Denizot F, Golstein P: Ctla-8, Cloned from an Activated T-Cell, Bearing Au-Rich Messenger-RNA Instability Sequences, and Homologous to a Herpesvirus Saimiri Gene. J Immunol 1993, 150(12):5445–5456. 8390535

[5] Krueger JG, Brunner PM: Interleukin-17 alters the biology of many cell types involved in the genesis of psoriasis, systemic inflammation, and associated comorbidities. Exp Dermatol 2018, 27(2):115–123. doi:10.1111/exd.13467:29152791

[6] Von Stebut E, Boehncke WH, Ghoreschi K, Gori T, Kaya Z, Thaci D, Schaffler A: IL-17A in Psoriasis and Beyond: Cardiovascular and Metabolic Implications. Front Immunol 2019, 10:3096. doi:10.3389/fimmu.2019.03096. 32010143 PMC6974482

[7] Brembilla NC, Senra L, Boehncke WH: The IL-17 Family of Cytokines in Psoriasis: IL-17A and Beyond. Frontiers in Immunology 2018, 9. doi:ARTN 168 10.3389/fimmu.2018.01682:PMC6088173 30127781

[8] Nadeem Ahmed SFA, Al-Harbi Naif O, Fardan Ali S, El-Sherbeeny Ahmed M, Ibrahim Khalid E, Attia Sabry M: IL-17A causes depression-like symptoms via NFκB and p38MAPK signaling pathways in mice: Implications for psoriasis-associated depression. Cytokine 2017(97):14–24. doi:doi: 10.1016/j.cyto.2017.05.018.:28570931

[9] Beurel E, Jope RS: Depression-like Behavior is Promoted by Inflammatory Th17 Cells in Mice. Biol Psychiat 2014, 75(9):401s–401s.

[10] Kim J, Suh YH, Chang KA: Interleukin-17 induced by cumulative mild stress promoted depression-like behaviors in young adult mice. Mol Brain 2021, 14(1). doi:ARTN 11 10.1186/s13041-020-00726-x:PMC7805143 33441182

[11] Griffiths CEM, Fava M, Miller AH, Russell J, Ball SG, Xu W, Acharya N, Rapaport MH: Impact of Ixekizumab Treatment on Depressive Symptoms and Systemic Inflammation in Patients with Moderate-to-Severe Psoriasis: An Integrated Analysis of Three Phase 3 Clinical Studies. Psychother Psychosom 2017, 86(5):260–267. doi:10.1159/000479163:28903122

[12] Zhu X, Sakamoto S, Ishii C : Dectin-1 signaling on colonic γδ T cells promotes psychosocial stress responses. Nat Immunol 2023, 24:625–636. 36941398 10.1038/s41590-023-01447-8 PMC10704545

[13] Zafiriou E DA, Siokas V, Tsigalou C, Dardiotis E, Bogdanos DP: Depression and Obesity in Patients With Psoriasis and Psoriatic Arthritis: Is IL-17-Mediated Immune Dysregulation the Connecting Link? Front Immunol 2021:12:699848. doi:10.3389/fimmu.2021.699848.:34367160 PMC8334867

[14] Wang L, Yi TS, Kortylewski M, Pardoll DM, Zeng DF, Yu H: IL-17 can promote tumor growth through an IL-6-Stat3 signaling pathway. J Exp Med 2009, 206(7):1457–1464. doi:10.1084/jem.20090207:19564351 PMC2715087

[15] Wu S, Rhee KJ, Albesiano E, Rabizadeh S, Wu X, Yen HR, Huso DL, Brancati FL, Wick E, McAllister F : A human colonic commensal promotes colon tumorigenesis via activation of T helper type 17 T cell responses. Nat Med 2009, 15(9):1016–1022. doi:10.1038/nm.2015. 19701202 PMC3034219

[16] Lan R, Moududee SA, Ge D, Wang A, Liu YZ, You Z: Blood Levels of Interleukin-17 Family Members in Healthy Individuals and Various Diseases. Serican Journal of Medicine 2026, 3(1):24398. doi:10.17161/sjm.v3i1.24938:

[17] Numasaki M, Fukushi J, Ono M, Narula SK, Zavodny PJ, Kudo T, Robbins PD, Tahara H, Lotze MT: Interleukin-17 promotes angiogenesis and tumor growth. Blood 2003, 101(7):2620–2627. doi:10.1182/blood-2002-05-1461:12411307

[18] Punt S, Langenhoff JM, Putter H, Fleuren GJ, Gorter A, Jordanova ES: The correlations between IL-17 vs. Th17 cells and cancer patient survival: a systematic review. Oncoimmunology 2015, 4(2). doi:ARTN e984547 10.4161/2162402X.2014.984547:PMC4404813 25949881

[19] Xiang T, Long H, He L, Han X, Lin K, Liang Z, Zhuo W, Xie R, Zhu B: Interleukin-17 produced by tumor microenvironment promotes self-renewal of CD133+ cancer stem-like cells in ovarian cancer. Oncogene 2015, 34(2):165–176. doi:10.1038/onc.2013.537:24362529

[20] Kim BS, Kuen DS, Koh CH, Kim HD, Chang SH, Kim S, Jeon YK, Park YJ, Choi G, Kim J : Type 17 immunity promotes the exhaustion of CD8(+) T cells in cancer. J Immunother Cancer 2021, 9(6). doi:10.1136/jitc-2021-002603. PMC8183213 34083422

[21] Chung AS, Lee J, Ferrara N: Targeting the tumour vasculature: insights from physiological angiogenesis. Nat Rev Cancer 2010, 10(7):505–514. doi:10.1038/nrc2868:20574450

[22] Zhang XR, Li BJ, Lan T, Chiari C, Ye XY, Wang KP, Chen J: The role of interleukin-17 in inflammation-related cancers. Frontiers in Immunology 2025, 15. doi:ARTN 1479505 10.3389/fimmu.2024.1479505:PMC11790576 39906741

[23] Liu S, Zhang Q, Chen C, Ge D, Qu Y, Chen R, Fan YM, Li N, Tang WW, Zhang W : Hyper-insulinemia enhances interleukin-17-induced inflammation to promote prostate cancer development in obese mice through inhibiting glycogen synthase kinase 3-mediated phosphorylation and degradation of interleukin-17 receptor. Oncotarget 2016, 7(12):13651–13666. doi:7296 [pii] 10.18632/oncotarget.7296:26871944 PMC4924668

[24] Jin B, Moududee SA, Ge D, Zhou P, Wang AR, Liu YZ, You Z: SCF(FBXW11) Complex Targets Interleukin-17 Receptor A for Ubiquitin-Proteasome-Mediated Degradation. Biomedicines 2024, 12(4). doi:10.3390/biomedicines12040755. PMC11047997 38672111

[25] Johnson JL, Yaron TM, Huntsman EM, Kerelsky A, Song J, Regev A, Lin TY, Liberatore K, Cizin DM, Cohen BM : An atlas of substrate specificities for the human serine/threonine kinome. Nature 2023, 613(7945):759–766. doi:10.1038/s41586-022-05575-3. 36631611 PMC9876800

[26] Lochhead PA, Sibbet G, Morrice N, Cleghon V: Activation-loop autophosphorylation is mediated by a novel transitional intermediate form of DYRKs. Cell 2005, 121(6):925–936. doi:10.1016/j.cell.2005.03.034:15960979

[27] Miyata Y, Nishida E: DYRK1A binds to an evolutionarily conserved WD40-repeat protein WDR68 and induces its nuclear translocation. Bba-Mol Cell Res 2011, 1813(10):1728–1739. doi:10.1016/j.bbamcr.2011.06.023:21777625

[28] You ZB, Dong Y, Kong XT, Zhang Y, Vessella RL, Melamed J: Differential expression of IL-17RC isoforms in androgen-dependent and androgen-independent prostate cancers. Neoplasia 2007, 9(6):464–470. doi:10.1593/neo.07109:17603628 PMC1899256

[29] Qian YC, Liu CN, Hartupee J, Altuntas CZ, Gulen MF, Jane-Wit D, Xiao JH, Lu Y, Giltiay N, Liu JB : The adaptor Act1 is required for interleukin 17-dependent signaling associated with autoimmune and inflammatory disease. Nature Immunology 2007, 8(3):247–256. doi:10.1038/ni1439:17277779

[30] Vankley H, Hale SM: Assay for Protein by Dye Binding. Anal Biochem 1977, 81(2):485–487. doi:Doi 10.1016/0003-2697(77)90725-4:907113

[31] Senevirathne C, Pflum MK: Biotinylated phosphoproteins from kinase-catalyzed biotinylation are stable to phosphatases: implications for phosphoproteomics. Chem-BioChem 2013, 14(3):381–387. doi:10.1002/cbic.201200626. PMC4524292 23335220

[32] Jin K, Ewton DZ, Park S, Hu J, Friedman E: Mirk regulates the exit of colon cancer cells from quiescence. J Biol Chem 2009, 284(34):22916–22925. doi:10.1074/jbc.M109.035519. 19542220 PMC2755699

[33] Zhou N, Yuan SD, Wang RC, Zhang WF, Chen JJ: Role of dual specificity tyrosine-phosphorylation-regulated kinase 1B (Dyrk1B) in S-phase entry of HPV E7 expressing cells from quiescence. Oncotarget 2015, 6(31):30745–30761. doi:10.18632/oncotarget.5222:26307683 PMC4741565

[34] Monin L, Gaffen SL: Interleukin 17 Family Cytokines: Signaling Mechanisms, Biological Activities, and Therapeutic Implications. Csh Perspect Biol 2018, 10(4). doi:ARTN a028522 10.1101/cshperspect.a028522:PMC5732092 28620097

[35] Goepfert A, Lehmann S, Blank J, Kolbinger F, Rondeau JM: Structural Analysis Reveals that the Cytokine IL-17F Forms a Homodimeric Complex with Receptor IL-17RC to Drive IL-17RA-Independent Signaling. Immunity 2020, 52(3):499–+. doi:10.1016/j.immuni.2020.02.004:32187518

[36] Li XX, Bechara R, Zhao JJ, McGeachy MJ, Gaffen SL: IL-17 receptor-based signaling and implications for disease. Nature Immunology 2019, 20(12):1594–1602. doi:10.1038/s41590-019-0514-y:31745337 PMC6943935

[37] Ma CM, Lin WL, Liu ZY, Tang W, Gautam R, Li H, Qian YC, Huang H, Wang XJ: NDR1 protein kinase promotes IL-17-and TNF-α-mediated inflammation by competitively binding TRAF3. EMBO Reports 2017, 18(4):586–602. doi:10.15252/embr.201642140:28219902 PMC5376972

[38] Zhao JJ, Chen X, Herjan T, Li XX: The role of interleukin-17 in tumor development and progression. J Exp Med 2020, 217(1). doi:ARTN e20190297 10.1084/jem.20190297:PMC7037244 31727782

[39] Liu C, Liu R, Wang B: Blocking IL-17A enhances tumor response to anti-PD-1 immunotherapy in microsatellite stable colorectal cancer (vol 9, e001895, 2021). Journal for Immunotherapy of Cancer 2021, 9(10). doi:ARTN e001895corr1 10.1136/jitc-2020-001895corr1:PMC7813395 33462141

[40] McGeachy MJ, Cua DJ, Gaffen SL: The IL-17 Family of Cytokines in Health and Disease. Immunity 2019, 50(4):892–906. doi:10.1016/j.immuni.2019.03.021:30995505 PMC6474359

[41] Levin SD: IL-17 Receptor Signaling: Ubiquitin Gets In On the Act. Sci Signal 2009, 2(92). doi:ARTN pe64 10.1126/scisignal.292pe64:19825826

[42] Qu FF, Gao HC, Zhu S, Shi PQ, Zhang YF, Liu Y, Jallal B, Yao YH, Shi YF, Qian YC: TRAF6-Dependent Act1 Phosphorylation by the IκB Kinase-Related Kinases Suppresses Interleukin-17-Induced NF-κB Activation. Mol Cell Biol 2012, 32(19):3925–3937. doi:10.1128/Mcb.00268-12:22851696 PMC3457531

[43] Draberova H, Janusova S, Knizkova D, Semberova T, Pribikova M, Ujevic A, Harant K, Knapkova S, Hrdinka M, Fanfani V : Systematic analysis of the IL-17 receptor signalosome reveals a robust regulatory feedback loop. Embo J 2020, 39(17). doi:ARTN e104202 10.15252/embj.2019104202:PMC7459424 32696476

[44] Bulek K, Liu CN, Swaidani S, Wang LW, Page RC, Gulen MF, Herjan T, Abbadi A, Qian W, Sun DX : The inducible kinase IKKi is required for IL-17-dependent signaling associated with neutrophilia and pulmonary inflammation. Nature Immunology 2011, 12(9):844–U861. doi:10.1038/ni.2080:21822257 PMC3282992

[45] Becker W: Emerging role of DYRK family protein kinases as regulators of protein stability in cell cycle control. Cell Cycle 2012, 11(18):3389–3394. doi:10.4161/cc.21404.:22918246 PMC3466549

[46] Li SS, Huang K, Xu C, Zhang H, Wang X, Zhang R, Lu Y, Mohan M, Hu C: DYRK1B phosphorylates FOXO1 to promote hepatic gluconeo-genesis. Nucleic Acids Res 2025, 53(8). doi:ARTN gkaf319 10.1093/nar/gkaf319:PMC12034038 40287828

[47] Ashford AL, Dunkley TPJ, Cockerill M, Rowlinson RA, Baak LM, Gallo R, Balmanno K, Goodwin LM, Ward RA, Lochhead PA : Identification of DYRK1B as a substrate of ERK1/2 and characterisation of the kinase activity of DYRK1B mutants from cancer and metabolic syndrome. Cell Mol Life Sci 2016, 73(4):883–900. doi:10.1007/s00018-015-2032-x:26346493 PMC4735261

[48] Zeng XH, Xu JQ, Liu JQ, Liu Y, Yang SQ, Huang JS, Fan CP, Guo MX, Sun GH: DYRK4 upregulates antiviral innate immunity by promoting IRF3 activation. EMBO Reports 2025, 26(3):690–719. doi:10.1038/s44319-024-00352-x:39702801 PMC11811199

[49] Bateman A, Martin MJ, Orchard S, Magrane M, Ahmad S, Alpi E, Bowler-Barnett EH, Britto R, Cukura A, Denny P : UniProt: the Universal Protein Knowledgebase in 2023. Nucleic Acids Res 2023, 51(D1):D523–D531. doi:10.1093/nar/gkac1052:36408920 PMC9825514

[50] Vorwerk VA, Wilms G, Babendreyer A, Becker W: Differential regulation of expression of the protein kinases DYRK1A and DYRK1B in cancer cells. Sci Rep-Uk 2024, 14(1). doi:ARTN 23926 10.1038/s41598-024-74190-1:PMC11471791 39397076

[51] Mercer SE, Ewton DZ, Deng XB, Lim S, Mazur TR, Friedman E: Mirk/Dyrk1B mediates survival during the differentiation of C2C12 myoblasts. Journal of Biological Chemistry 2005, 280(27):25788–25801. doi:10.1074/jbc.M413594200:15851482 PMC1201501

[52] Khor B, Gagnon JD, Goel G, Roche MI, Conway KL, Tran K, Aldrich LN, Sundberg TB, Paterson AM, Mordecai S : The kinase DYRK1A reciprocally regulates the differentiation of Th17 and regulatory T cells. eLife 2015, 4. doi:ARTN e05920 10.7554/eLife.05920:PMC4441007 25998054

